# Orbital Intradiploic Epidermoid Cyst: A Case Report of a Rare Entity

**DOI:** 10.7759/cureus.52072

**Published:** 2024-01-10

**Authors:** Fahad Albadr, Hamdan S Aldosari, Naif S Alsaber, Abdulaziz S Aljurayyad, Wejdan Shabi, Saba M Aldusaymani

**Affiliations:** 1 Radiology and Medical Imaging/Neuroradiology, King Saud University Medical City/College of Medicine, King Saud University, Riyadh, SAU; 2 Medicine, King Saud University Medical City/College of Medicine, King Saud University, Riyadh, SAU; 3 Medicine, AlMaarefa University, Riyadh, SAU

**Keywords:** intradiploic epidermoid cyst, orbital tumors, orbital bone, ent procedures, neuroradiology, neurosugery, frontal bone cyst, orbital epidermoid cyst, giant epidermoid cyst, congenital epidermoid cyst

## Abstract

Cranial epidermoid cysts are relatively rare. More frequently reported in middle-aged men with a wide variety of signs and symptoms such as headache, seizures, cerebellar and cranial nerve deficits/visual disturbance. The approach for surgical removal of the cyst depends on its size and location.

In addition, a multidisciplinary team must be involved due to the common occurrence of misdiagnosis. We present the unusual age of presentation for intradiploic epidermoid cysts. A 14-year-old boy is complaining of a 2-month history of painless progressive swelling of the right eyebrow. Magnetic resonance imaging revealed an intradiploic cystic mass within the right frontal bone. The cystic mass was removed, and histological examination confirmed the diagnosis of an epidermoid cyst. This case illustrated the potential of developing intradiploic epidermoid cysts in pediatrics.

## Introduction

Cranial epidermoid cysts are a relatively infrequent occurrence, accounting for around 0.2% to 1% of all cerebral malignancies [1]. 25% of these cases are intradiploic epidermoid tumors, more frequently reported in middle-aged men. Epidermoid cysts occur due to the inability of the surface ectoderm to properly detach from the underlying tissues, as well as the sequestration or implantation of the surface ectoderm. Additionally, they can be categorized as congenital epidermoid cysts, which occur due to the implantation of ectoderm during the closure of the neural groove or other fusion lines of epithelial tissue. On the other hand, secondary or acquired epidermoid cysts are typically generated by the inclusion of surface epithelium following a traumatic event [2]. They are typically slow-growing and can cause marked distortion of the underlying brain without producing many neurological signs. Depending on their size and location, intradiploic epidermoid cysts of the orbital bone may remain asymptomatic for a long time or cause various symptoms. Common symptoms include proptosis, pain, seizures, headaches, and visual disturbances caused by compression either on the eye globe or the brain [3]. Although epidermoid cysts are benign, malignant transformation is increasing in several reported cases, ranging from 6 months to 33 years from initial diagnosis to malignant change. However, the true incidence and risk factors of malignant transformation remain unknown, and thus, treatment is often guided by the location and the presence of symptoms [4]. Squamous cell carcinoma can develop from the malignant transformation of epidermoid and dermoid cysts into either an extant benign cyst or a remnant of a previously removed lesion [5]. Two annual visits are recommended for follow-up after the cyst is surgically excised, ensuring effective removal to prevent recurrence or malignant transformation [4]. The diagnosis of an intradiploic epidermoid cyst should be correlated with the patient's clinical symptoms, radiological findings, and histopathological examination to establish a definitive diagnosis and rule out other differential diagnoses such as dermoid cysts, arachnoid cysts, and mucoceles [6]. Here, we report a case of an intradiploic epidermoid cyst involving the right frontal bone in a 14-year-old boy.

## Case presentation

**Figure 1:**
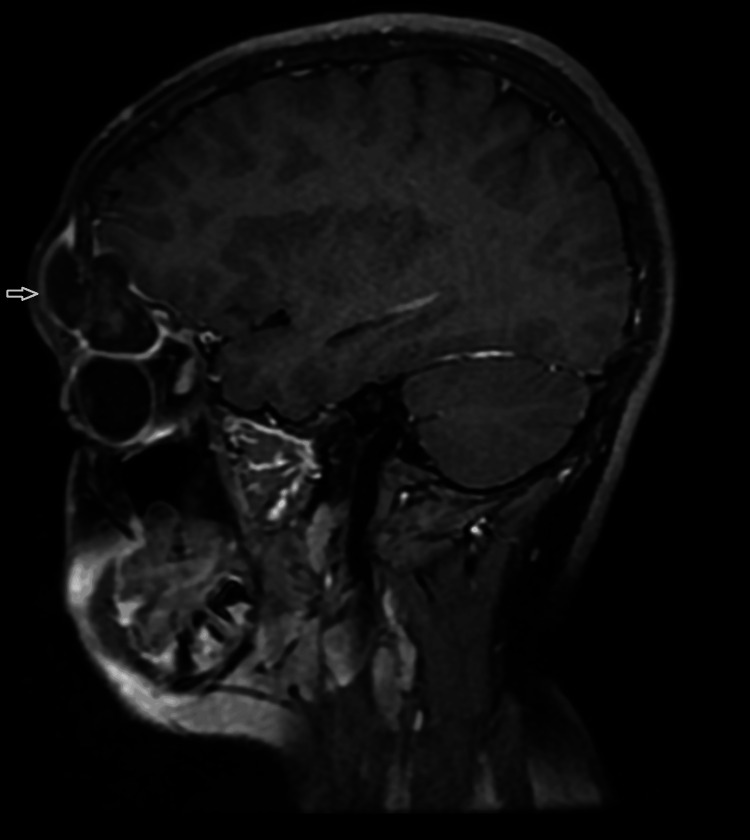
Post-contrast image demonstrates peripheral wall enhancement without enhancement of the internal component

**Figure 2:**
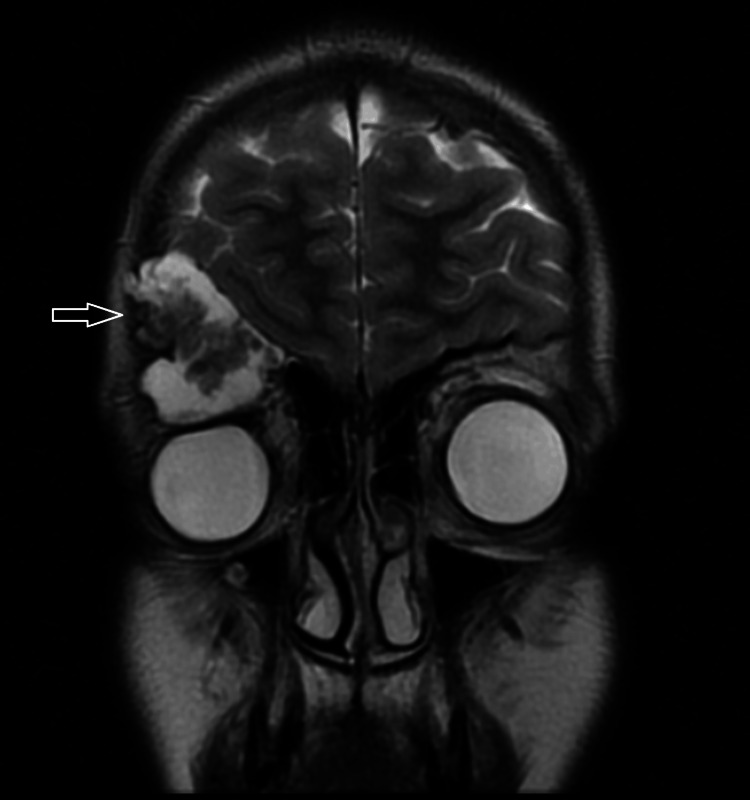
T2-weighted image shows right supero-lateral intraorbital 2.0x1.0x0.5 cm osseous expansile lobulated cystic lesion with internal soft tissue component exerting mass effect over the right globe downward as well as thinning of the superior orbital bone without intracranial or intraocular component.

**Figure 3:**
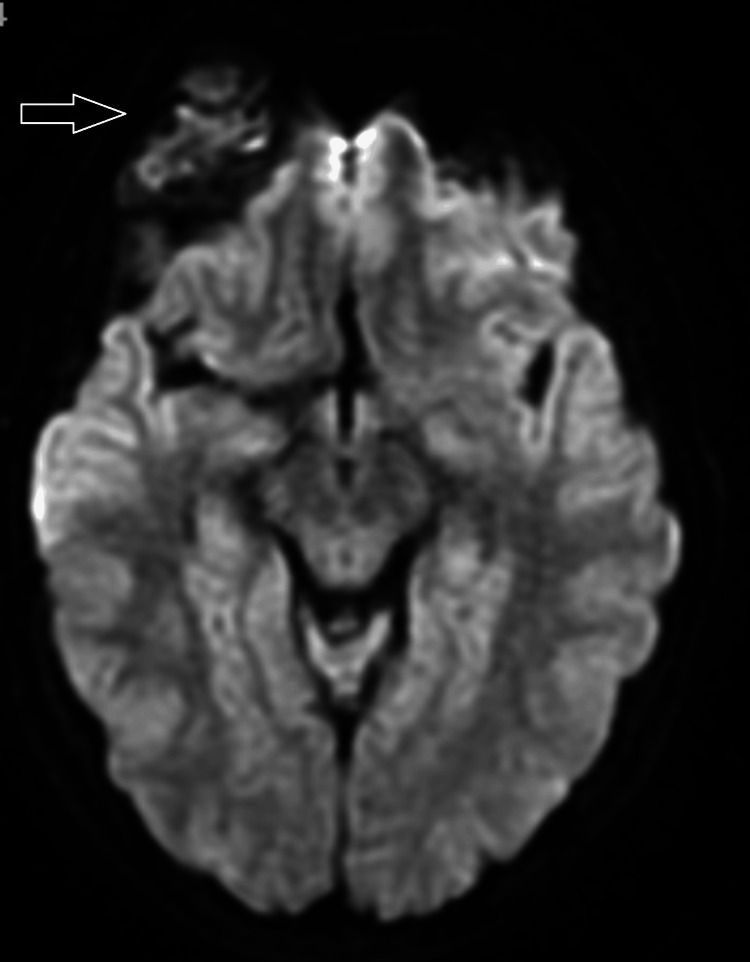
Diffusion-weighted image shows heterogeneous diffusion restriction within the central aspect of the lesion.

A 14-year-old boy from low socioeconomic status presented to the ER with a two-month history of small, gradually progressive, painless swelling over the forehead just above the right eyebrow. The swelling was first noticed 2 months before ER presentation; it was small then rapidly enlarged, causing severe headache and episodic vertigo accompanied by intermittent and transient vision loss in both eyes lasting a few seconds with no known precipitating factors. The patient denied any history of trauma, radiation, seizures, vomiting, and drowsiness. Besides having sinusitis and an abscess managed with antibiotics in the past, he did not have any other medical problems. The general examination of swelling revealed a dome-shaped firm skin-colored nodule above the right eyebrow, measuring 2x2 cm. The ophthalmic examination revealed right eye ptosis, limited right eye abduction and elevation, and right eye monocular diplopia, especially on the right lateral and up gaze. Apart from this, the findings of the neurological examination were unremarkable. MRI scans were performed during preoperative planning (Figure 1-3).

**Figure 4:**
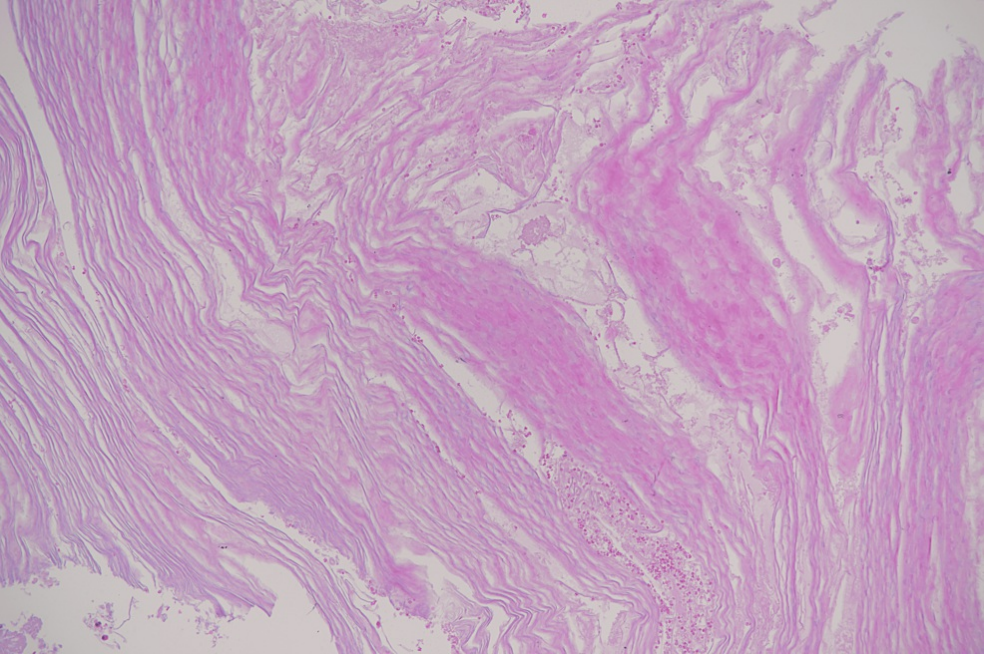
Laminated keratin flakes with foci of acute inflammatory cells. No epithelium was identified (H&E x 10)

The radiological differential diagnoses included intradiploic epidermoid cyst, eosinophilic granuloma, dermoid cyst, giant cell tumor, and plasmacytoma. The patient was referred to the neurosurgery department for craniotomy. Later, a craniotomy was performed, and surgical resection of the cyst was done. Histopathological findings confirmed the diagnosis of an epidermoid cyst (Figure 4).

**Figure 5:**
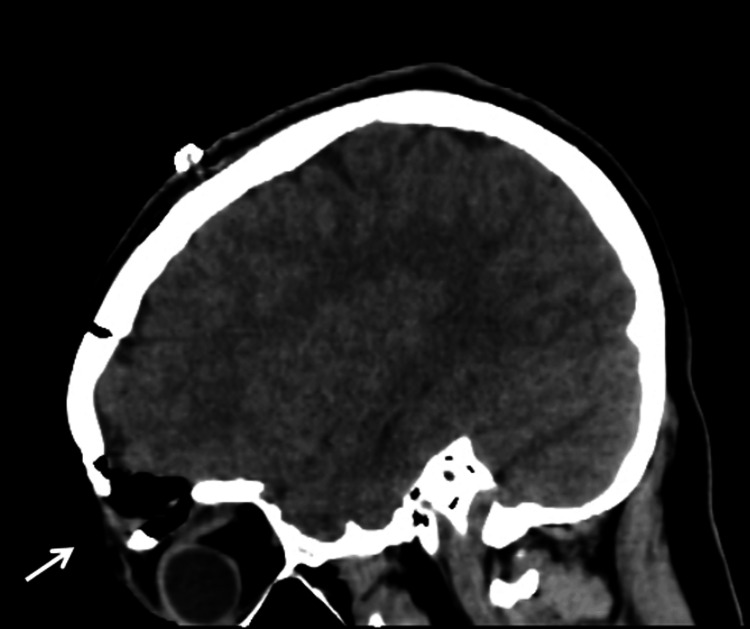
CT scan of the brain without contrast, post-resection of the lesion demonstrates expected post-operative changes (arrow) without immediate complication

Post-operatively, the patient improved significantly and resolved his signs and symptoms completely. Post-operative CT was done to ensure complete removal of the cyst (Figure 5).

## Discussion

Intradiploic orbital epidermoid cysts are rare pathologies occurring within the diploe of the cranium. These are composed of keratinaceous simple squamous epithelia without adjacent skin appendages; the presence of the latter represents the histopathological hallmark of dermoid cysts [7]. The presentation may be incidental or that of insidious chronic headaches with or without exophthalmos and ptosis [8]. The prevalence of intracranial epidermoid cysts is estimated to be less than 0.3 to 2%, 25% of which are reportedly intradiploic in nature [2]. Nearly 200 cases of intradiploic epidermoid cysts have been reported in the literature, and orbital intradiploic forms likely comprise less than 20% of such cases; however, the precise prevalence remains unclear [7,8]. We hereby report one additional case with an unusual age of presentation; the core presentation was consistent with previous reports involving the orbital bone [9]. The patient's clinical course was uncomplicated with successful removal, and the patient's clinical course was uncomplicated with physical and radiological examination, ensuring effective and complete cyst removal.

While a diagnosis can often be suspected clinically, imaging modalities are indispensable to diagnose and therapeutically plan the excision of these masses. Sonography, CT, and MRI are the diagnostic modalities of choice for cystic intradiploic lesions. Using sonography helps in the identification of cystic features within a lesion. Nevertheless, when employing high-frequency ultrasound transducers, epidermoid cysts can present as hypoechoic, resembling solid structures. Differential diagnosis among lipoma, dermoid, and epidermoid cysts can be achieved by noting density values in the range of fat. Detecting small calcified regions is more effective using CT scans rather than ultrasound [10].

We narrowed our differentials based on radiological findings and presentation. Dermoid cysts are most often diagnosed at an early age and are typically located along the midline, with fat content, which appears as a hypodense lesion on CT and might be accompanied by calcifications and hyperintense on T1WI. Eosinophilic granulomas, commonly found in children, appear as hypodense lesions on CT, hypointense on T1WI, hyperintense on T2WI/FLAIR, and they appear on Diffusion-weighted images. Giant cell tumors may contain radiopaque components; MRI images of this lesion reveal fluid levels throughout the cystic component [11]. Plastocytoma mainly occurs in adults and shows contrast enhancement on MRI; they appear as a lytic lesion or small soft tissue masses on CT, hypointense on T1WI, and hyperintense on T2WI/FLAIR [12].

Despite the challenge of differentiating the mass from other osteolytic cranial tumors, our radiological diagnosis conclusively identified it as an intradiploic epidermoid cyst. The diagnosis was made by considering the patient's age and the radiological findings, histopathological findings, restricted diffusion on MRI, the lack of dural invasion, and the absence of contrast enhancement [13]. MRI plays a significant role due to its multiplanar capabilities and superior soft tissue contrast, providing essential insights into these lesions' content, extent, and spatial association [10].

Furthermore, a diffusion-weighted image is highly valuable in distinguishing between CSF spaces and primary and post-operative residual epidermoid cysts, and brain tissue [14]. True restriction with a low apparent diffusion coefficient value in intradiploic epidermoid cysts is due to the restricted motion of the protons within the cyst and the presence of cholesterol and cell debris. [13].

Intracranial epidermoid cysts are usually benign, however, cases of malignant transformation have been reported to occur even as late as 12 years following the initial presentation [15]. We suggest additional studies to identify the risk factors and the best treatment options for intracranial epidermoid cysts. Treatment of intradiploic orbital epidermoid cysts typically involves surgical excision to prevent unwanted complications and ensure proper management [15]. Fortunately, in our case, the patient did not develop complications post-operatively, and all the symptoms resolved completely.

## Conclusions

Epidermoid cysts are benign lesions arising from ectodermal tissue typically found in the skin or subcutaneous tissue. However, Intradiploic epidermoid cysts of the orbital bone are rare and occur more frequently in middle-aged men; our case is a 14-year-old boy. Epidermoid cyst necessitates evaluation and management by a multidisciplinary team of ophthalmologists, neurosurgeons, pathologists, and radiologists.
